# Double burden of malnutrition among migrants and refugees in developed countries: A mixed-methods systematic review

**DOI:** 10.1371/journal.pone.0273382

**Published:** 2022-08-18

**Authors:** Alex Ankomah, Judith Byaruhanga, Emma Woolley, Sheila Boamah, Blessing Akombi-Inyang

**Affiliations:** 1 School of Population Health, University of New South Wales, Sydney, Australia; 2 Obstetrics and Gynaecology Team, Airport Women’s Hospital, Accra, Ghana; 3 School of Medicine and Public health, The University of Newcastle, Callaghan, Australia; 4 School of Education, Macquarie University, Sydney, Australia; 5 School of Nursing, McMaster University, Hamilton, Canada; Sreenidhi Institute of Science and Technology, INDIA

## Abstract

Migrants and refugees living in high income countries (HICs) have an increased risk of developing non-communicable diseases (NCDs) due to malnutrition at different stages over their life course. This systematic review aims to examine the double burden of malnutrition (DBM) among migrants and refugees in developed countries. This review was informed by the standard Preferred Reporting Items for Systematic Reviews and Meta-Analyses (PRISMA 2020) guidelines. Eligible studies were peer-reviewed observational studies that focused on the DBM in refugee or migrant populations; and were written in English with full texts available and accessible. A combination of keywords was used to search 8 electronic bibliographic databases including Ovid MEDLINE, EMBASE, PsycINFO, CINAHL, ProQuest, Scopus, PubMed, and web of science. There is a paucity of research into the DBM among migrants and refugees residing in HICs. Of a total of 2344 articles retrieved from eight databases, 5 studies met the inclusion criteria. All included studies showed the co-existence of some form of undernutrition and overnutrition within the same population. Overnutrition (overweight and obesity) in the studied populations ranged from 11.1% to 42% while undernutrition (stunting, wasting and underweight) ranged from 0.3% to 17%. Standard measures for anthropometry as determined by World Health Organization (WHO) were used for data collection in all included studies. A piloted form informed by the Cochrane Public Health Group Data Extraction and Assessment Template was used in the extraction of data from retrieved studies. Quality assessment of included studies was performed using the study assessment tools of the National Heart, Lung, and Blood Institute of the National Institutes of Health (NIH) and showed that all studies were of fair quality. Interventions that simultaneously target more than one form of malnutrition especially amongst migrant and refugee populations must be implemented for policy solutions to be effective. This review has been registered by the PROSPERO international prospective registry for systematic reviews, reference CRD42020192416.

## Introduction

In recent decades, the forces of globalisation, demographic change, income and economic growth and urbanisation has largely influenced the nutrition landscape and resulted in a significant global shift in the quality and quantity of human diets and nutrition-related epidemiology [1, 2]. The shift in the global nutrition landscape ushered in the double burden of malnutrition (DBM) which is the coexistence of various forms of malnutrition within individuals, households, and communities over the life course [2, 3]. This phenomenon is rapidly emerging as a major public health concern especially within migrant and refugee populations in developed countries [2, 3].

Migrants and refugees undergo the complex and dynamic [4] process of acculturation which denotes the process by which minority groups adopt the culture of their host country [5]. In most cases, this adoption is associated with a shift from a predominance of one form of malnutrition in home country to another in the host country [6]. The medical, developmental, economic, and social consequences of such shift in nutritional status are significant for migrants and their families, as well as for the host countries [7]. The DBM amongst migrants and refugees in developed countries may be enabled by multiple acculturative, environmental, and socio-economic factors [8, 9] relating to limited income, unemployment, poor access to health services, clean water as well as limited food and humanitarian assistance [7]. Though these enablers have been identified [8, 9], yet limited studies have synthesised evidence on the DBM among migrant and refugee populations living in high income countries (HICs) to guide targeted interventions given the potential negative impact it exerts on both the migrants and the health system of host countries.

Previous studies have solely reported undernutrition among migrant communities [10, 11] while others have provided evidence for the growing burden of overnutrition [12–14]. To address DBM, actions that simultaneously target more than one form of malnutrition especially amongst migrant and refugee populations given their unique circumstance must be implemented for policy solutions to be effective [2]. Therefore, this systematic review examines DBM among migrants and refugees originating from LMICs living in HICs.

## Methods

This systematic review adheres to the standard Preferred Reporting Items for Systematic Reviews and Meta-Analyses (PRISMA) guidelines [15]. The PRISMA 2020 statement includes a checklist with 27 essential items for ensuring reporting transparency. This review has been registered by the PROSPERO international prospective registry for systematic reviews, reference CRD42020192416 and protocol published by Social Science Protocols [16].

### Outcomes of interest

The primary outcome of this study is the DBM which is the coexistence of undernutrition along with overweight and obesity, micro-nutrient deficiency or diet-related noncommunicable diseases, within individuals, households, and populations, and across the life course [1]. According to World Health Organization (WHO), undernutrition has four broad sub-forms which include (i) stunting (low height-for-age) and an indicator of linear growth retardation and cumulative growth deficits in children; (ii) wasting (low weight-for-height) which measures body mass in relation to height and describes current nutritional status; and (iii) underweight (low weight-for-age) which is a composite index of height-for-age and weight-for-height. This systematic review focuses on children with a Z-score below minus two standard deviations (− 2 SD) from the median of the World Health Organisation (WHO) reference population [17, 18]. Overnutrition develops from an abundant intake of nutrients, leading to excessive weight and obesity (high weight-for-height) [19]. Micro-nutrient deficiency occurs as a result of deficiency in essential minerals and vitamins needed by the body in small quantities [19]. Diet-related NCDs include cardiovascular diseases such as heart attacks and stroke, and often linked with high blood pressure, certain cancers, and diabetes.

### Participants

There were no limits to the age, gender, social status, or ethnicity of participants. The target population for this review were migrants and refugees residing in HICs. A migrant is defined as a person who moves away from his or her place of usual residence, whether within a country or across an international border, temporarily or permanently, and for a variety of reasons [20]. A refugee is a person who is outside his country of nationality or habitual residence based on a well-founded fear of persecution because of his race, religion, nationality, membership in a particular social group or political opinion, and is unable or unwilling to avail himself of the protection of that country, or to return there, for fear of persecution [21].

### Eligibility criteria

Studies were included in the review if they were (i) peer-reviewed observational studies (cross-sectional studies, cohort studies and case-control studies); (ii) focused on the DBM in refugee or migrant populations; and (iii) written in English with their full texts available and accessible. There was no restriction with the publication year as all studies which reported the DBM were eligible. The research team does not have the logistical and financial capacity to search for, retrieve and translate literature published in languages other than English. In addition, to searching peer reviewed literature, a search of grey literature including publications from key institutions, organisations and government websites was also conducted.

### Search strategy

A comprehensive search was conducted using appropriate search terms, subject heading truncations (*), and Boolean operators ("AND", "OR" and "NOT") which were modified to adhere to the specifications of each database being searched. Eight electronic bibliographic databases were searched including Ovid MEDLINE, EMBASE, PsycINFO, CINAHL, ProQuest, Scopus, PubMed, and web of science. The following combination of search terms and keywords was used in the search:

Migrant* OR immigrant* OR emigrant* OR expat* OR refugee* OR asylum seeker* OR displaced person*

AND

Malnutrition OR Undernutrition OR Undernourish* OR Stunting OR Wasting OR Underweight OR Overnutrition OR Obesity OR Overweight OR Micronutrient deficiency OR Vitamin A deficiency OR Vitamin D deficiency OR Iodine deficiency OR Iron deficiency OR Anaemia OR Non-communicable diseases OR NCD OR Diabetes OR Cancer OR Hypertension OR Cardiovascular diseases

AND

Developed countries OR High-income countries OR Host countries OR United States OR United Kingdom OR Europe OR Australia OR OECD countries.

The reference lists of eligible studies were reviewed and articles that met the inclusion criteria were retained. The search strategy was first implemented in July 2021 and a re-run of the strategy was conducted in February 2022 prior to the final analysis.

### Study selection

Studies retrieved from the various databases were imported into the Covidence software and duplicates were removed. This was followed by the screening of the titles and abstracts to determine relevance. Thereafter, full texts of the remaining studies were read for eligibility and studies that met the inclusion criteria were retained. The entire screening process was undertaken independently by two researchers and disagreements were resolved through discussion and consensus. A search log was kept for accountability and transparency. A PRISMA flowchart is used to present the study selection [22].

### Data extraction

A piloted form informed by the Cochrane Public Health Group Data Extraction and Assessment Template was used in the extraction of data from retrieved studies. Data extracted include author, year of publication, country of study, study design and setting, study participants, data collection methods, and primary outcome measures (DBM). Data extraction was conducted by one author (J.B.) and independently reviewed by a second author (B.A.). The two authors perused the reference lists of the retained studies to identify additional relevant studies.

### Quality assessment

Quality assessment involved the analysis of the methodological quality of the retained studies. The quality of the eligible studies was assessed using the study assessment tools of the National Heart, Lung, and Blood Institute of the National Institutes of Health (NIH) for quality assessment of Observational Cohort and Cross-Sectional Studies [23]. The NIH checklist measures 14 criteria to assess the external validity (based on potential selection bias) and internal validity (based on potential measurement biases and confounding) of eligible studies. Studies were considered as ‘good’ if they met 10–14 criteria, ‘fair’ if they met 5–9 criteria and ‘poor’ if they met ≤4 criteria as shown in S1 Table. A high-quality rating implies a low risk of bias and vice versa [23]. Emerging evidence has suggested that the NIH checklist is a robust tool for assessing risk of bias in observational studies [24, 25].

## Results

**Fig 1:**
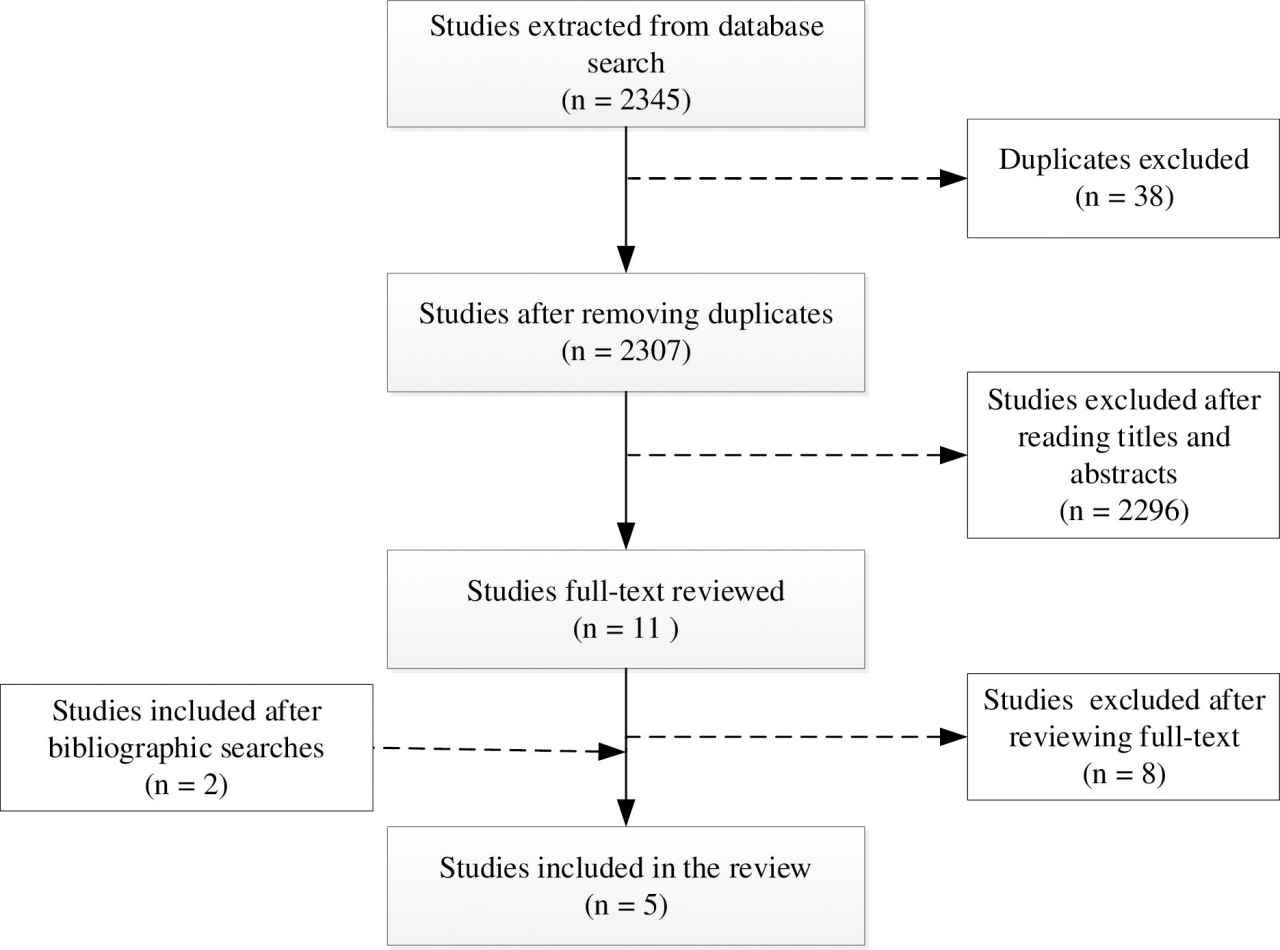
PRISMA flowchart for the selection of eligible studies.

A total of 2345 studies were retrieved from 8 databases. After the removal of 38 duplicates, 2307 studies were retained for further screening. Screening of the titles and abstracts resulted in the exclusion of 2296 studies. The full-text of the remaining 11 studies were retrieved and assessed for eligibility, which led to a further exclusion of 8 studies. Only 5 studies met the inclusion criteria. A manual search of the bibliographic references of the retained studies identified 2 additional studies thereby giving a total of 5 studies as shown in Fig 1.

### Characteristics of included studies

Table 1 shows a summary of the studies included in this review. The studies were conducted in 5 different HICs namely USA [26], Switzerland [27], Greece [28], Australia [29] and Mexico [30]. The number of participants in the retained studies varied from 177 to 1767. The participants’ age ranged from 0 to 21 years. Three studies reported a higher proportion of males to females [27, 28, 30]. Four of the studies were cross-sectional [26, 28, 29], while 2 study used mixed methods [27, 30]. Standard measures for anthropometry were used for data collection in all included studies. Three studies examined malnutrition among children and adolescents [26, 29, 30] while 1 study was among children under 5 years [28] and 1 study among adults [27]. Quality assessment of included studies shows that all studies were of fair quality. All included studies were published between 2006 and 2021.

**Table 1:** Summary of selected studies.

Author (Year) Country [Ref]	Study design and setting	Study characteristics	Data collection	Primary outcomes	Quality Assessment
Esmaili *et al*. (2021) USA [26]	Retrospective Cross-sectional study Paediatric Refugee Patients	Participants: 327 children/adolescents Age: 0–21 years Females: 53% Males: 47%	Retrospective electronic medical record review using the Epic Maestro Care database	Stunting (17%), Overweight (21%), Vitamin D deficiency (79%) and anemia (13%)	6 Fair
Amstutz *et al*. (2020) Switzerland [27]	Mixed methods study Hospital consultations, health centres and refugees’ housing sites.	Participants: 354 adults Age: 31.3 ± 9.6 Females: 32% Males: 68%	Anthropometric measurements and face-2-face interviews	Underweight (5.7%) and overweight (42%)	7 Fair
Walpole *et al*. (2018) Greece [28]	Cross-sectional study Syrian refugee Camps	Participants: 177 children Age: < 5 years Females: 49% Males: 51%	Anthropometric measurements entered into the WHO Anthro software	Overweight (11.1%), Wasting (1.9%), Stunting (17%)	6 Fair
Renzaho *et al*. (2006) Australia [29]	Cross-sectional study Sub-Saharan African refugees and migrants	Participants: 337 children Age: 3–12 years Females: 51% Males: 49%	Anthropometric measurements	Overweight (18.4%), obesity (8.6%), wasting (4.3%), underweight (1.2%) and stunting (0.3%)	7 Fair
Jiménez-Cruz *et al*. (2003) Mexico [30]	Mixed methods study Primary school in Tijuana	Participants: 1767 children/adolescents Age: 5–14 years Females: 623 Males: 644	Anthropometric measurements and conducting interviews	Overweight and Obesity (38%), Underweight (1.2%), Stunting (4.8%)	6 Fair

### Summary of findings

The findings from the included studies suggest that both undernutrition and overnutrition may be observed within migrant and refugee populations in HICs. Prevalence of overnutrition (overweight and obesity) in the studied populations ranged from 11.1% to 42% while undernutrition (stunting, wasting and underweight) ranged from 0.3% to 17%. Esmaili [26] while exploring the health needs, trends of health care utilization, and barriers to care of a diverse population of refugee children resettled in North Carolina found a high prevalence of nutritional problems, including growth stunting (17%), overweight (21%), Vitamin D deficiency (79% of the 39 tested), and anaemia (13%). Amstutz [27] investigated the prevalence of undernutrition and obesity among refugees in Geneva and reported prevalence of underweight at 5.7% and overweight at 42%. Walpole [28] carried out a growth assessment to inform appropriate nutritional support for child refugees in Greece and found a prevalence of wasting (1.9%), stunting (17%) and overweight (11.1%). Renzaho [29] assessed the anthropometric status of migrant and refugee children from sub-Saharan Africa in Australia and reported overweight (18.4%), obesity (8.6%), wasting (4.3%), underweight (1.2%) and stunting (0.3%). Jiménez-Cruz [30] assessed the prevalence of overweight, obesity, and undernutrition among migrant children. The study reported an overall prevalence of overweight and obesity as 38%, underweight as 1.2%, and stunting as 4.8%. The 14 criteria used to evaluate the quality of included studies showed that all studies were of fair quality. The details of domain-specific score are provided in S1 Table.

## Discussion

The present paper examines the DBM among migrants and refugees in developed countries and reports the presence of both undernutrition and overnutrition in the same population. This review also identified a paucity of high-quality studies on DBM within migrant and refugee populations who have moved from LICs to HICs. This result raises challenging issues towards the design of migrant support programmes, as well as future prevention and provision of care for nutrition-associated co-morbidities among migrant and refugee populations.

The finding from this review is consistent with that of a systematic review conducted among Western Sahara Refugees in Algeria which reported the double burden of obesity and under-nutrition in Western Sahara refugees households [31]. It has also been found that growth among migrants and refugees is accelerated exponentially in a new environment owing to acculturation and increasing prevalence of unhealthy behaviours [3, 4]. This is particularly prevalent among migrants and refugees from Sub-Saharan Africa and Asia who might have initially undergone growth challenges due to the limited availability of food in home country. Thus, indicating that migrating to a high-income host country is likely to increases the risk of overweight, obesity and/or micronutrient deficiency in migrants and refugees. This can be attributed to an abundance of food, and an environment which discourages physical activity and healthy diets, while promoting sedentary activities and consumption of unhealthy foods and beverages in host country [2].

The DBM among migrants and refugees in developed countries reported in this study could be due to changes in post-migration nutrition as a result of changes in the food marketing industry. Though the impacts of these changes are not limited to migrant and refugee sub-populations, however, these sub-population tend to be more vulnerable to its impacts due to their unique sociocultural dispositions which could be influenced by acculturative forces. Recent evidence suggests that changes in the food marketing industry have resulted in a decline in the number of fresh markets globally as well as an increase in access and consumption of ultra-processed foods especially among migrant and refugee populations [1, 32]. This new nutrition reality has resulted in major changes in the nutritional status of vulnerable subpopulations globally thereby, causing major nutritional challenges [6]. Ultra-processed foods have been found to play a significant role in the onset of obesity and non-communicable diseases, hence, driving the overnutrition arm of the DBM [33].

The rising rate of overnutrition seen among refugees and migrants may, therefore, heavily be influenced by the increasing purchase of affordable and high-caloric ultra-processed meals which replaced home-cooked meals in these communities. One driver for increased consumption of ultra-processed foods among migrants and refugees in developed countries may be the lack of ability to use local seasonal produce. One study identified in this review found that while fruit and vegetables were often affordable in the host country, they were different to the types of fruits and vegetables that were available in their home country, and a lack of ability or confidence to cook with these available foods presented as a barrier to healthy eating [27]. In addition, ultra-processed foods may present as a cheap, reliable alternative for families experiencing financial pressures.

This review highlights the need for more research examining DBM in HICs among refugee and migrant populations. Addressing overweight and obesity in migrant and refugee populations in HICs requires tailored approaches, alongside policies and strategies to address the obesogenic environment. The findings of this study will offer insights to policymakers around targeted public health interventions to tackle the DBM within migrant and refugee populations in HICs. This study will also inform future research efforts by identifying gaps and strengths in effective interventions targeting the needs of migrants and refugees in developed countries. Finally, findings from this review could further inform settlement and health policies and practice for migrants and refugees. In addition, this review also highlights the need for more research examining DBM in HIC among refugee and migrant populations.

This study is a comprehensive search of the existing literature on the DBM among migrant and refugee populations residing in HICs. It applied a systematic and transparent approach to data synthesis to enable reproductivity of study findings. However, this study had some limitation despite its strengths. First, qualitative studies were not included as studies selected were restricted to quantitative studies. The inclusion of qualitative studies in systematic reviews enables triangulation of findings or offer alternative explanations [34]. Second, this review excluded studies not published in English. Exclusion of such studies could lead to missing key literature generated by non-English-speaking researchers and regional organisations.

## Conclusions

Although the DBM has been shown to be an evolving global health issue, there is a paucity of evidence of the prevalence and impact of the DBM in migrant and refugee populations in HICs. The burden and impact of the combination of undernutrition, particularly in early life, with a transition to the often-obesogenic environment of a HIC is poorly understood and requires further investment to target policy and interventions to improve health within this vulnerable subpopulation.

## Supporting information

S1 Table Quality assessment of included studies. (PDF)

S2 Table PRISMA 2020 checklist. (PDF)
